# The Effects of the COVID-19-induced Lockdown on the Social Capital and Cultural Capital in Italy

**DOI:** 10.1007/s11205-023-03140-7

**Published:** 2023-06-01

**Authors:** Elisa Menardo, Marta Viola, Alice Bacherini, Luana Angelini, Roberto Cubelli, Giulia Balboni

**Affiliations:** 1 Department of Human Sciences, University of Verona, Verona, Italy; 2 Department of Social and Developmental Psychology, Sapienza University of Roma, Roma, Italy; 3 Department of Philosophy, Social Sciences and Education, University of Perugia, Piazza G. Ermini, 1, Perugia, 06123 Italy; 4 Department of Psychology and Cognitive Sciences, University of Trento, Rovereto, Italy

**Keywords:** Structural equation models, Cluster analysis, Sociocultural level, Socioeconomic status, Pandemic

## Abstract

The present study investigated the effects of the first COVID-19 lockdown on the Cultural and Social Capitals in Italy in a large group of adults (*n* = 1125). The relationships between the COVID-19 spread and participants’ Cultural Capital, Social Capital, educational level, occupational prestige, and age were studied using structural equation models. For women but not for men, pandemic spread was positively affected by occupational prestige and it had a positive relationship with their Social Capital (women: CFI = 0.949; RMSEA = 0.059 [CI = 0.045-0.075]; men: CFI = 0.959; RMSEA = 0.064 [CI = 0.039–0.087]). Moreover, the participants were divided into three validated clusters based on their Cultural and Social Capitals levels to investigate changes in the Capitals compared with the pre-lockdown period. It was found that the lockdown contributed to improving the gap among individuals increasing high levels and decreasing low levels of both the Capitals. People with high Cultural and Social Capitals seemed to have seized the opportunity given by COVID-19 restrictions to cultivate their cultural interests and become more involved within their networks. In contrast, individuals with low Cultural and Social Capitals paid the highest price for the social isolation. Given that the Capitals encourage healthy behavior and influence well-being and mental health, institutions should develop or improve their policies and practices to foster individual resources, and make fairer opportunities available during the pandemic.

**Supplementary Information:**

The online version contains supplementary material available at 10.1007/s11205-023-03140-7.

## Introduction

The COVID-19 pandemic is one of the most serious and widespread health emergencies. Worldwide, over 750 million cases and over 6.8 million deaths have been registered (World Health Organization, February 22, 2023). To counteract the spread of the virus, restrictive measures have been adopted by almost all national governments. On March 9, 2020, two months after the China Center for Disease Control and Prevention identified the new coronavirus named Sars-nCoV-2, the Italian government declared a lockdown that lasted until May 18, 2020. During this period, 208,009 cases were recorded, mainly in northern Italy (Riccardo et al., 2020). All commercial activities and services considered non-essential remained closed, including places for cultural production and consumption, and all citizens were forced to stay indoors. The lockdown inevitably led to changes in people’s living habits, and caused unemployment, loneliness, and inadequate access to essential goods (Gallè et al., 2022). Presumably, these changes impacted people’s resources, including Cultural Capital and Social Capital.

Cultural Capital refers to the knowledge and use of cultural codes relevant to the community in which people live (Bourdieu & Passeron, 1970; Lamont & Lareau, 1988). Cultural Capital includes three dimensions (Balboni et al., 2019): cultural activities, such as visiting museums and attending musical events or theater performances, and goods (books or artworks) (e.g., Dumais 2002); cultural technical skills and knowledge, such as using foreign languages and technology, performing in concerts, plays, or shows, creating artworks (e.g., Lareau & Weininger 2003); and engagement with cultural, community service, religious, or political groups and associations (e.g., Jeannotte 2003).

Social Capital refers to the set of actual or potential resources associated with durable and trustworthy social network connections (Bourdieu, 1986). Social Capital includes collective/community Social Capital and individual/personal Social Capital (e.g., Villalonga-Olives & Kawachi 2017). Collective Social Capital, sometimes called Civic Capital (Bartscher et al., 2021; Fazio & Lavecchia, 2013; Guiso et al., 2011), refers to resources shared within a community to overcome problems (e.g., blood donation, trust in others; Durante et al., 2021). Personal Social Capital regards individual resources (wealth, education, social status, and political power) associated with social network connections that an individual can use to reach benefits (Chen et al., 2009; Liu et al., 2016). In this paper, we focused on personal Social Capital, which includes two types of social ties (Chen et al., 2009; Kreuter & Lezin, 2002). Bonding Social Capital refers to the resources associated with networks within homogeneous groups that share interests and mutual attraction, such as family, neighbors, friends, and colleagues; bridging Social Capital refers to resources associated with networks in the community, such as groups or associations (economic, social, cultural, recreational, religious, and political).

Studies have shown that Cultural Capital and Social Capital are inter-related (Menardo, Viola et al., 2022) and that they encourage healthy behavior, influence well-being and mental health (Giordano & Lindstrom, 2011; Menardo et al., 2017; Pellicci et al., 2015; Talmage et al., 2022). Both Capitals provide critical resources that support people during a crisis such as a pandemic: for example, dissemination of health information and promotion of access to local health services (Chen & Meng, 2015), social distance compliance (Barrios et al., 2021), reciprocal trust between people and between people and government (Harring et al., 2021). The Capitals reflect resources that help increase community resilience (Norris et al., 2008; Paarlberg et al., 2020).

The Capitals are likely to have undergone changes during the COVID-19 pandemic. Millions of people were being forced to stay indoors for extended periods, that lead to social isolation and radically changed daily routines. This may have had several consequences. While social relationships were inevitably affected, social isolation may have fostered the development of a strong sense of community and network. The lockdown greatly affected the daily practices of working and spending free time as well. Some people may have found alternative ways to communicate, maintain relationships, have fun, and be involved in cultural activities (Gu & Huang, 2022). In contrast, other people may have lost many social contacts and cultural opportunities because of the pandemic.

Studies on Social Capital were primarily focused on the relationship between Social Capital and the diffusion of COVID-19 and have shown a general tendency toward less diffusion of COVID-19 in areas with high Social Capital (e.g., Alfano 2022; Bartscher et al., 2021; Bylok, 2022). Some findings are consistent with the positive relationship between civil Social Capital and adherence to health policy protocols (Bartscher et al., 2021; Borgonovi et al., 2021), which is also found in pre-COVID-19 studies (Aldrich, 2012; Nakagawa & Shaw, 2004). However, different dimensions of personal Social Capital appear to have different effects. Bonding Social Capital has resulted related to fewer COVID-19 cases (Alfano, 2022), which may be attributed to more assistance and mutual support, typical of high-bonding communities (Fraser & Aldrich, 2021). Bridging Social Capital, on the other hand, appears to have played a protective role only in the early stages of the pandemic (Fraser & Aldrich, 2021). When the virus was not very widespread, the amount of knowledge and information circulating in high-bridging communities helped people cope with worsening situations (Teng & Takemoto, 2022). On the contrary, in the more advanced pandemic stages, participation in groups and trust in them were associated with higher mortality (Elgar et al., 2020).

Few studies have investigated Cultural Capital during the pandemic, although the cultural sector is one of the most affected (Causi, 2021). The results were inconsistent, some showing an increase (Artuso & Palladino, 2021), some a decrease (Codagnone et al., 2021), and some showed the same patterns of pre-pandemic engagement of Cultural Capital, although the delivery of cultural consumption shifted toward online and digital modes (Causi, 2021; Feder et al., 2021). Interestingly, increasing patterns of Cultural Capital were found among individuals who were already highly engaged, showing higher cultural consumption (Feder et al., 2021), and increased existing inequalities (Bennett & Silva, 2006). These results suggest that during the pandemic access to cultural offerings was declined for the most vulnerable population (Modernel & Cornejo, 2022).

In sum, the literature suggests that both Cultural Capital and Social Capital have been affected by the COVID-19 pandemic (e.g., Elgar et al., 2020; Modernel & Cornejo, 2022). However, there are still some open research questions.

In the present study, first, we investigated whether and how the relationship between Cultural Capital and Social Capital changed in a pandemic context and how the COVID spread was associated with this relationship. For this aim, a previous structural equation model tested in a non-pandemic context (Menardo, Viola et al., 2022) was replicated during the first lockdown in Italy in a large group of adults. As in the previous model, we considered the relationships between Cultural Capital and Social Capital, educational level and occupational prestige as socioeconomic status indicators, and age in men and women separately. In addition, we added the COVID-19 spread in the areas where the participants lived during the first lockdown. Unlike the previous works on COVID-19, we considered both Cultural Capital and Social Capital with their subdimensions (i.e., consuming, participating, and expert using Cultural Capital; and bonding and bridging Social Capital), and verified the goodness of introducing non-linear relationships between age and the other variables.

Second, we compared the Cultural Capital and Social Capital levels shown during the lockdown with those pre-pandemics. We investigated whether any changes in both Cultural and Social Capitals were related to the levels of and the relationship between them during the lockdown. Indeed, previous studies showed different patterns of change depending on the underlying social and cultural resources (Artuso & Palladino, 2021; Feder et al., 2021; Fraser & Aldrich, 2021). For this purpose, we divided the participants into clusters based on the levels and relationships of Cultural Capital and Social Capital during the lockdown. We then validated the obtained clusters by comparing them to externally known results: We verified that they were also different on variables, such as educational level, occupational prestige, and gender, which were independent of the clustering but known as related to Cultural Capital and Social Capital (e.g., Menardo, Viola et al., 2022; Nakai, 2011; Van Groenou & Van Tilburg, 2003). Finally, we investigated whether the clusters of participants differed in the changes shown during the lockdown. In this way, we can verify if the changes in Cultural Capital and Social Capital during the lockdown have varied according to the different levels and the relationship between them.

The meeting point between the two aims is the relationship between Cultural and Social Capitals. First, we wanted to verify whether the association between the two forms of capital (and with socioeconomic indicators) is different from those observed in the pre-pandemic period. Secondly, we wanted to verify if, during a pandemic period, it is possible to identify different relationship patterns between Cultural and Social Capitals (i.e., low-low, low-high, high-high) and if these different patterns are associated with the pre-pandemic level of Capitals.

## Method

### Instruments

#### Cultural Capital

An updated version of the Scale of Cultural Capital (Balboni et al., 2019) was used to measure Cultural Capital that may be developed also online. It is composed of 14 items with a 5-point Likert scale (0 to 4) to investigate the three main dimensions of Cultural Capital: (1) *Participating* in community service, political, religious, and cultural groups/associations (four items); (2) *Consuming* cultural products and events, such as visiting museums and attending/watching theater performances (six items); (3) *Expert using* technical skills such as reading books or e-books for study or work and using foreign languages (four items). The score ranges from 0 to 4 for each item (Balboni et al., 2019). The factorial structure of the updated version of the Scale of Cultural Capital was verified via confirmatory factor analysis (CFA) with 466 adults (69% women) aged 19–67 years (mean [*SD*] = 36.63[14.44]) with a high school diploma (50%) or a degree (47%). Good goodness-of-fit indices were achieved: rCFI = 0.909, rRMSEA = 0.060 [CI = 0.050–0.70], and SRMR = 0.052.

In the present study, for each item, participants were invited to consider only the lockdown period: for example, “How many times during the lockdown period did you attend/watch a theatre performance from the beginning to the end, whether in person, on television, or online (for example, YouTube, Netflix)?”. After each item, participants were required to specify how much the described behavior had changed compared to the pre-lockdown period, according to a 3-point Likert scale: 1 = *decreased*, 2 = *unchanged*, and 3 = *increased.* The reliability for the total scale, and for participating, consuming, and expert using dimension scores was slightly low (McDonald’s omega = 0.75, 0.56, 0.51, and 0.67, respectively).

#### Social Capital

We used the Personal On-Offline Social Capital Brief Scale (Menardo, Cubelli et al., 2022), developed by selecting the 16 most informative items from the Italian version of the Personal Social Capital Scale (Chen et al., 2009; Wang et al., 2014). The Personal On-Offline Social Capital Brief Scale measures the two Social Capital bonding and bridging dimensions through four composite items each, which included 11 and eight items, respectively, evaluated on a 5-point Likert scale (1 to 5). The first four composite items (11 items) assess offline and online bonding Social Capital with questions on the size, trust and support received, and quality/resources (broad connections and high reputation/influence) of informal networks (i.e., friends, work/study colleagues, people in the neighborhood, and online contacts). The remaining four composite items (8 items) evaluate offline and online bridging Social Capital with questions on the number, rights/interests represented, support received, and qualities/resources (broad social connections and extensive social influence) of community service, cultural, religious, and political groups/associations (including those with online activities). The rejected items of the Personal Social Capital Scale concerned resources associated with family members, relatives, and fellow citizens or old childhood friends/old classmates (bonding Social Capital), and recreational and economic/professional associations/groups (bridging Social Capital). The Personal On-Offline Social Capital Brief showed excellent validity and reliability (Menardo, Cubelli et al., 2022).

In the present study, for each item, participants were invited to consider only the lockdown period: e.g., “Among your friends, how many did definitely support you upon your request or would have done if you had asked them during the lockdown period”. Again, after each item, participants were required to specify how much the described behavior had changed compared to the pre-lockdown period, according to a 3-point Likert scale: 1 = *decreased*, 2 = *unchanged*, and 3 = *increased.* The reliability of the scores in the present study was adequate (McDonald’s omega was 0.76, 0.69, and 0.70, for the total scale, bonding, and bridging scores, respectively).

#### Socioeconomic Status

Educational level (i.e., years of education) and occupational prestige were used to measure socioeconomic status (e.g., Coscarelli et al., 2007). Occupational prestige refers to the public perception of an individual’s social standing based on the position of their occupation in the social stratification and was measured using the International Cambridge Scale (ICAMS; Meraviglia et al., 2016). It associates a score (range 29.48–65.07) of occupational stratification at each occupation as classified according to the International Standard Classification of Occupations (ISCO-88, International Labour Office 1990). Low scores represent inferior positions (low-prestige occupations).

#### Social Desirability

The Balanced Inventory of Desirable Responding (BIDR-6)-Short Form Italian version (Bobbio & Manganelli, 2011; Paulhus, 1991) consists of 16 items rated on a 6-point Likert scale to measure the unconscious tendency to provide honest but positively biased responses, as well as the habitual and conscious presentation of a favorable public image. Individuals with a total score exceeding the 95th centile of the normative sample were identified as the simulators. This scale has been reported to show adequate reliability and validity (Bobbio & Manganelli, 2011). In the present study, McDonald’s omega was 0.63.

#### Questions on COVID-19 Lockdown

Questions were developed concerning the COVID-19 lockdown (March 9–May 18, 2020) to investigate whether individuals stayed where they generally lived, with whom and, eventually, with how many adults.

#### COVID-19 Spread

**Fig. 1:**
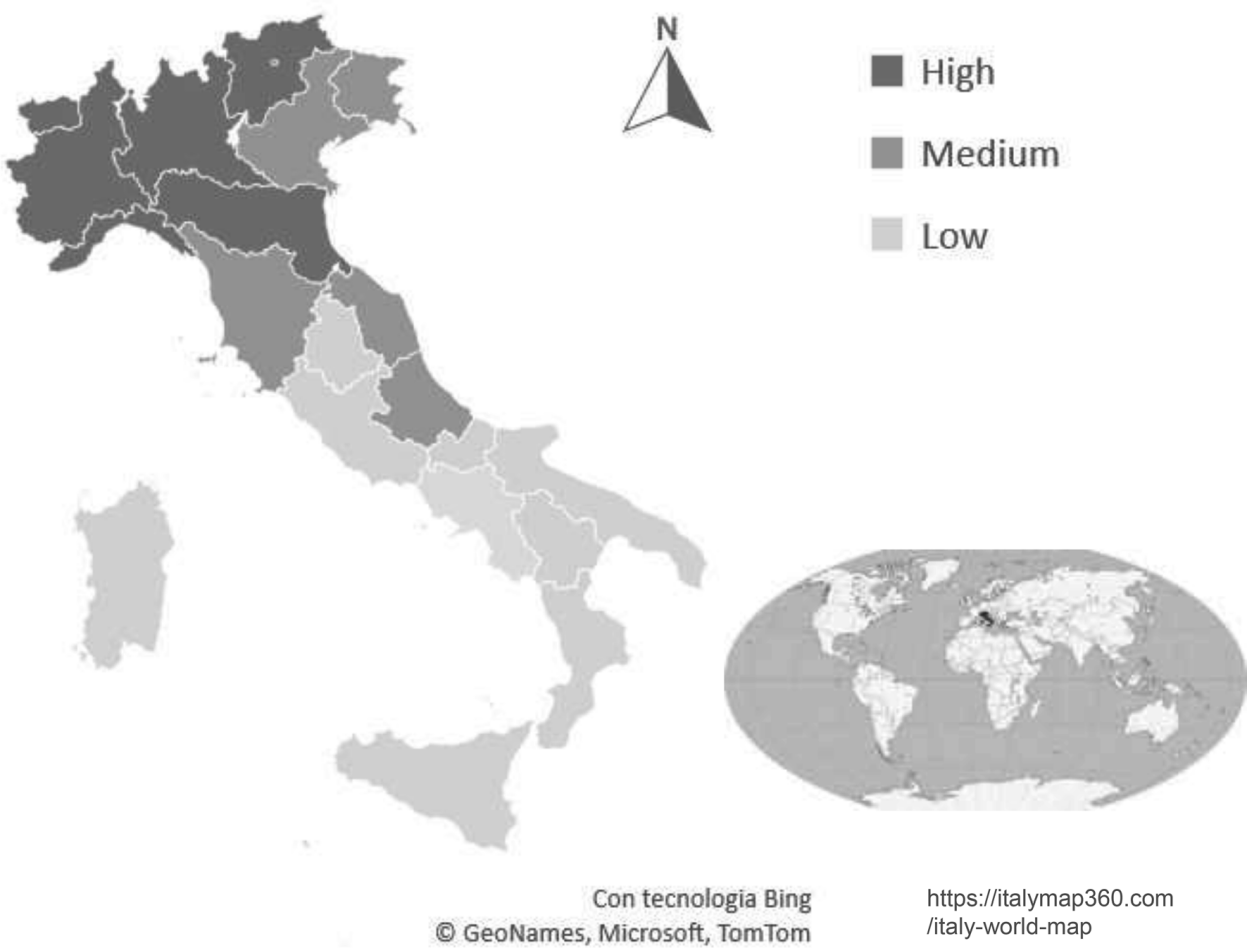
COVID-19 Spread in Italy during the first lockdown in March-May 2020 (Data from Istituto Nazionale di Statistica, 2020a)

Data on the COVID-19 spread in Italy during the first lockdown (March-May 2020) were retrieved from a government source (Istituto Nazionale di Statistica, 2020a). Italian regions were classified as having a low COVID-19 spread, if there were less than 150 cases per 100,000 residents; medium, if there were 150 to 450 cases per 100,000 residents; and high, if there were more than 450 cases per 100,000 residents (see Fig. 1). For each participant, the COVID-19 spread in the area of domicile during the lockdown was classified as low, medium, and high, in agreement with the data of the corresponding region.

**Table 1:** Characteristics of All Participants, Men, and Women

	Total (*n* = 1,125)	Men (*n* = 389)	Women (*n* = 736)
Age (years)			
Mean (*SD*)	38.20 (13.93)	38.83 (14.55)	37.87 (13.59)
Range	18–74	18–73	18–74
Educational Level (years)			
Mean (*SD*)	15.71 (3.20)	15.43 (3.17)	15.85 (3.2)
Range	5–26	8 − 25	5–26
Occupational Prestige (score range: 21.07–85.27)			
Mean (*SD*)	57.00 (13.91)	55.94 (14.11)	57.56 (13.77)
Range	23.43–82.71	23.43–82.71	23.43–82.71
COVID-19 Spread in the Area of Domicile (%)			
Low	24	25	23
Medium	51	51	51
High	25	24	26

### Participants

The participants included 1,125 Italian adults (65% women) aged 18–74 years who, during the lockdown, stayed in the northern (33%), central (49%), and southern (18%) regions of Italy. In Table 1, age, educational level, occupational prestige, and the COVID-19 spread are reported for the total sample, and for men and women, separately.

A total of 1,550 individuals agreed to participate in the online survey. Of these, 27 did not complete the questionnaire and were excluded. Of the remaining 1,523 individuals, 58 were excluded because they scored above the cutoff on the social desirability scale BIDR-6 (Bobbio & Manganelli, 2011), thus indicating a simulation attempt. Furthermore, 306 individuals were excluded because they never had an occupation (e.g., housewives or students, *n* = 289), or their occupational prestige score was unavailable (i.e., military, *n* = 17) on the ICAMS (Meraviglia et al., 2016). Finally, in agreement with Tabachnick and Fidell’s (2013) suggestions on the assumptions to verify before using the structural equation models, 34 univariate outliers were excluded (see below).

The participants were not selected randomly but based on voluntary responses. Compared to the Italian population (Istituto Nazionale di Statistica, 2020b), there were no gender differences (*χ*^*2*^_(1)_ = 3.47, *p* = .06), but they were younger (*z* = -7.62, *p* < .001, Cohen’s *d* = 0.52). Moreover, participants resided more frequently in central Italy (*χ*^*2*^_(1)_ = 42.05, *p* < .001, Cohen’s *w* = 0.93) and less frequently in southern Italy (*χ*^*2*^_(1)_ = 7.53, *p* < .001, Cohen’s *w* = 0.65), whereas no differences were found for northern Italy (*χ*^*2*^_(1)_ = 3.67).

### Procedure

Data were collected from May 6, 2020 to June 3, 2020 immediately after the first lockdown in Italy. An online questionnaire, including all the instruments, was generated using Google Forms. Two versions of the questionnaire were available to counterbalance the order of the Scale of Cultural Capital and the Personal On-Offline Social Capital Brief Scale (CC-SC in 51% of cases and SC-CC in 49% of cases). Finally, the BIDR-6 and demographic questions were placed at the end.

Trained researchers who were unaware of the aims of the study disseminated the online questionnaire via popular Facebook and Telegram groups and the Instagram pages of popular persons, groups, and associations. The following services/institutes located in the overall area of Italy were reached: cultural, community service, religious, political, and recreational groups/associations; public and private schools and universities; student dorms; protective services; shops; and factories. The same written instructions were provided to all the participants.

### Data Analysis

#### Preliminary Analysis

Following Tabachnick and Fidell’s (2013) suggestions, the presence of univariate outliers (e.g., participants with a *z-*value higher than |3.29|) and multivariate outliers (e.g., participants for whom the probability associated with the Mahalanobis distance was lower than 0.001) was checked for all interval observed variables (i.e., age, educational level, occupational prestige, dimensions of Cultural Capital and Social Capital). The normality of the univariate distribution was verified by computing the asymmetry and kurtosis values, considering as appropriate the indices in the range of -1.00 − 1.00. Normality of the multivariate distribution was verified using the Mardia test.

#### Structural Equation Modeling: Identification of the Relationships Between Cultural Capital, Social Capital, Socioeconomic Status, Age, and COVID-19 Spread in the Domicile Area

The structural equation modeling allows verifying if a hypothesis set of multivariate causal or covariate relationships between latent and/or observed variables may explain and apply to a measured set of variables (e.g., Tabachnick & Fidell 2013).

Based on the results of a previous study in a non-pandemic context (Menardo, Viola et al., 2022), a structural equation model was run separately for men and women. Age was introduced as an observed variable that predicted the two interrelated observed variables educational level and occupational prestige. These, in turn, predicted the two interrelated latent variables Cultural Capital and Social Capital, which were measured with the corresponding dimensions as observed variables. The relationships between COVID-19 spread in the areas where individuals stayed during the lockdown and their educational level, occupational prestige, Cultural Capital, and Social Capital were also introduced into the model. The relationships between age and Cultural and Social Capitals, educational level and occupational prestige were modeled after checking the best function that described them.

The goodness of fit of the model was investigated using the R package Lavaan (Rosseel, 2012) via the maximum likelihood (ML) estimator. The goodness-of-fit indices were the chi-square statistic (*χ*^*2*^), comparative fit index (CFI), root-mean-square error of approximation (RMSEA) with associated 95% confidence intervals (CI), and standardized root-mean-square residual (SRMR) (Schermelleh-Engel et al., 2003). Values higher than 0.95 for CFI, smaller than 0.05 for RMSEA, and smaller than 0.08 for SRMR suggest a reasonable fit (Hu & Bentler, 1999; Schermelleh-Engel et al., 2003). The reported models are the final models after a stepwise removal strategy of the statistically nonsignificant paths until only statistically significant paths remained (and modification indices suggested no relevant modifications). The parameters reported were standardized covariance coefficients for the covariance relationships and structural coefficients for the causal relationships between variables.

Three alternative nested models were investigated and compared to the chosen model. These models were obtained by introducing one latent variable that was measured via the observed variables educational level and occupational prestige (Model A1), a second-order latent variable for Cultural Capital and Social Capital latent variables (Model A2), and both couples of introduced variables (Model A3). To this end, Akaike’s Information Criterion (AIC) was generated, with a lower value indicating a better-fitting model (Schermelleh-Engel et al., 2003).

#### Cluster Analysis: Identification of the Optimal Solution of Subgroups Based on their Cultural Capital and Social Capital Levels During the Lockdown

The cluster analysis aims to identify subgroups of participants so that participants in the same group are similar while participants in different groups are not similar (e.g., Milligan & Hirtle 2003).

A cluster analysis was run to identify the meaningful subgroups of participants with the minimum within and maximum between differences in the level Cultural Capital and Social Capital during the lockdown. We performed a two-stage hierarchical cluster analysis using the R package cluster (Maechler et al., 2022) and NbClust (Charrad et al., 2014). In the first stage, we determined the number of clusters based on the cluster centroids, that is, a point in space that represents a cluster and corresponds to the midpoint of the points of the cluster itself. Following Clatworthy et al. (2005) and Milligan and Sokol (1980), we used Ward’s minimum variance clustering method and the squared Euclidean distance as a measure of similarity. A mix of informal and formal rules was used to identify the optimal cluster solution: dendrogram’s examination, pseudo-*F* and pseudo *t*^*2*^ statistics, and theoretical interpretability of the final cluster solution. The pseudo-*F* is the ratio of between-cluster variance to within-cluster variance, and provides a measure of how separated the clusters are. The cluster solution with the higher value is the best solution. Pseudo *t*^*2*^ quantifies the difference in the ratio of the between-cluster variance to the within-cluster variance, when clusters are merged at a given step. If there is a distinct jump in *t*^*2*^ with X number of clusters, then X + 1 represents the optimal number of clusters. Moreover, to be reasonably representative, all clusters may be composed of at least 10% of the total participants. We repeated the first-stage cluster analysis using a different clustering method, that is, the complete linkage method, to formally check the reliability of the original Ward’s solution.

In the second stage, we used the k-means iterative partitioning procedure with the cluster centroids obtained using the first-stage Ward solution as the starting seed point to correct for improper initial assignment of participants to the cluster. This procedure is an iterative algorithm that refines the subdivision of the participants in each cycle to minimize the variance within the clusters and maximize the variance between them. To check the stability of the cluster solution, we randomly divided the participants into two subgroups and repeated the analyses.

#### Comparisons of the Clusters of Participants: Validation of the Cluster Solution and Investigation of Differences in the Changes in Cultural Capital and Social Capital During the Lockdown

To check the external validity of the cluster solution, we used ANOVA and Chi-square statistics to verify that the clusters were also different on variables, such as educational level, occupational prestige, and gender, not introduced for clustering, but related to Cultural Capital and Social Capital (e.g., Menardo, Viola et al., 2022; Nakai, 2011; Van Groenou & Van Tilburg, 2003).

To investigate the differences between the clusters in terms of changes in Cultural Capital and Social Capital shown during the lockdown, we also used ANOVA. Finally, to verify that any obtained differences in the Cultural and Social Capitals changes were independent of variables concerning the lockdown, the Chi-square statistic was used to compare the obtained clusters on COVID-19 spread in the area where the individual stayed, usual domicile or not, living situation, and number of adults.

In agreement with Cohen’s criteria (1988), effect sizes were evaluated as negligible (partial *ƞ*^2^ < 0.01; *d* < 0.20), small (0.01 ≤ partial *ƞ*^2^ < 0.06; 0.20 ≤ *d* < 0.50), medium (0.06 ≤ partial *ƞ*^2^ < 0.14, 0.50 ≤ *d* < 0.80), or large (partial *ƞ*^2^ ≥ 0.14, *d* ≥ 0.80).

## Results

### Preliminary Analysis

Thirty-four univariate outliers were excluded from the analysis. Multivariate outliers were not identified. All observed interval variables were normally distributed (skewness and kurtosis were between approximately − 1 and + 1). The Mardia’s index was equal to 12.7 for men and 13.9 for women, lower than the critical value of 15 (equal to *k*(*k* + 2) with *k* = 3), suggesting that the data were multivariate normally distributed. Table 2 shows the mean (*SD*) scores for the Scale of Cultural Capital and the Personal On-offline Social Capital Brief Scale for all participants, and for men and women, separately.

**Table 2:** Means (*SD*) Scores for the Scale of Cultural Capital and the Personal On-Offline Social Capital Brief Scale for All Participants, Men, and Women

	Total (*n* = 1,125)	Men (*n* = 389)	Women (*n* = 736)
Cultural Capital dimensions			
Participating (score range: 0–4)			
Mean (*SD*)	0.53 (0.69)	0.54 (0.70)	0.53 (0.69)
Range	0.00–2.75	0.00–2.75	0.00–2.75
Consuming (score range: 0–4)			
Mean (*SD*)	1.10 (0.62)	1.08 (0.64)	1.11 (0.62)
Range	0.00–3.17	0.00–3.17	0.00–3.17
Expert using (score range: 0–4)			
Mean (*SD*)	1.25 (0.65)	1.21 (0.65)	1.26 (0.65)
Range	0.00–3.25	0.00–3.25	0.00–3.25
Social Capital dimensions			
Bonding (score range: 1–5)			
Mean (*SD*)	3.15 (0.57)	3.11 (0.58)	3.18 (0.57)
Range	1.33–4.92	1.71–4.71	1.33–4.92
Bridging (score range: 1–5)			
Mean (*SD*)	2.96 (0.76)	2.88 (0.77)	3.01 (0.75)
Range	1.00–5.00	1.00–5.00	1.00–5.00

### Structural Equation Modeling: Identification of the Relationships Between Cultural Capital, Social Capital, Socioeconomic Status, Age, and COVID-19 Spread in the Domicile Area

The best function describing the relationships between age and Cultural Capital, Social Capital, educational level, and occupational prestige, was the cubic function (“U-shape”) (see Table S1 in the supplementary information). In particular, we found an inverse pattern across genders for the association between age and Cultural and Social Capitals. Middle-aged men had lower Cultural Capital and higher Social Capital than younger and older men. In contrast, middle-aged women had higher Cultural Capital and lower Social Capital than younger and older women. Both middle-aged men and women had lower educational levels and occupational prestige than younger and older men and women. The cubic function was then used to describe the association between age and these variables.

**Fig. 2:**
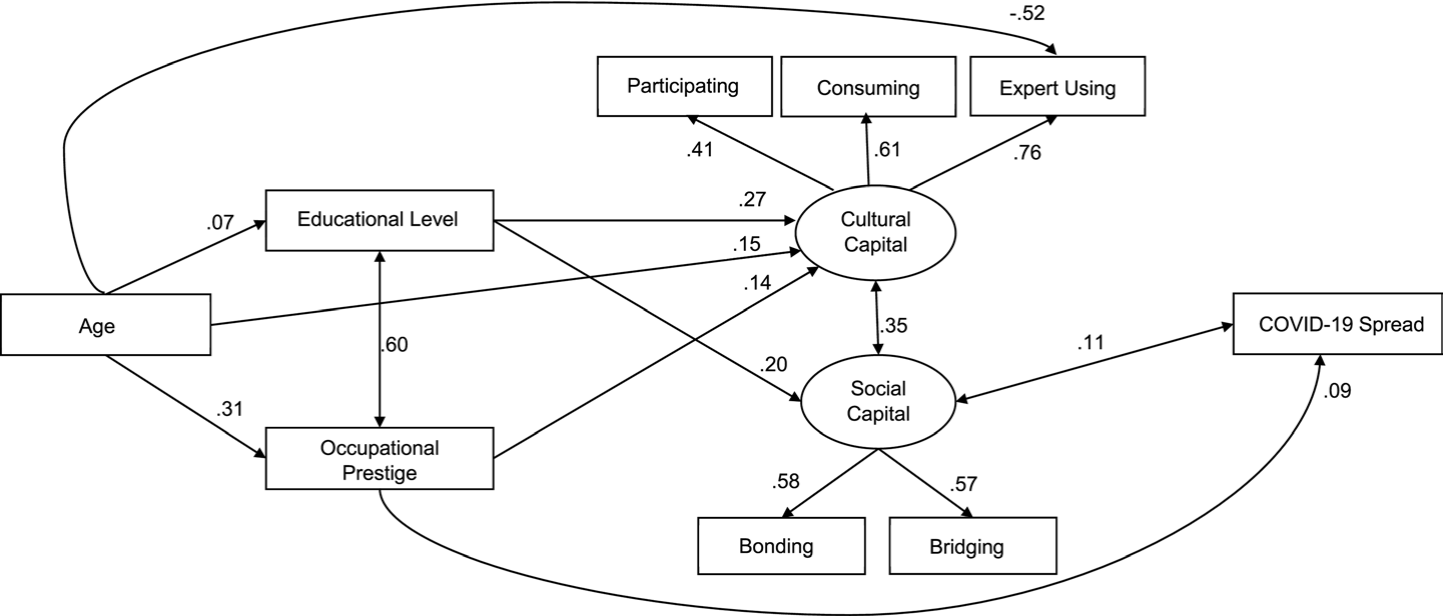
Structural equations model of the relationships between age, educational level, occupational prestige, Cultural Capital, Social Capital, and COVID-19 spread in women (*n *= 736). Values reported are the standardized covariance coefficients (lines with arrows at both ends) and structural coefficients (lines with one arrow) between variables

The resulting model presented good fit indices for women (*n* = 736): CFI = 0.949; RMSEA = 0.059 [CI = 0.045–0.075]; SRMR = 0.037; *χ*^*2*^_(19)_ = 68.41, *p* < .001; AIC = 21,452. As shown in Fig. 2, both Cultural Capital and Social Capital were well measured by their dimensions (i.e., participating, consuming, and expert using Cultural Capital and bonding and bridging Social Capital). Age positively predicted educational level, occupational prestige, and Cultural Capital but negatively predicted the expert using Cultural Capital dimension. Educational level and occupational prestige positively correlated and positively affected Cultural Capital. Educational level also positively affected Social Capital. Cultural Capital and Social Capital were positively correlated. The COVID-19 spread in the area where the participant stayed was positively predicted by occupational prestige and correlated with Social Capital. Age also had an indirect positive effect through occupational prestige on Cultural Capital (*β* indirect = 0.04; *SE* = 0.007; *p* = .018) and COVID spread (*β* indirect = 0.03; *SE* = 0.003; *p* = .018). The explained variance of Cultural Capital was 18% and that of Social Capital was 4%.

Concerning the alternative models, those with a second-order latent variable for Cultural Capital and Social Capital showed similar fit indices (CFI = 0.952; RMSEA = 0.056 [CI = 0.041 − 0.071]; SRMR = 0.036; *χ*^*2*^_(20)_ = 66.17, *p* < .001; AIC 21,448). In contrast, the other alternative models had worse goodness indices (see Table S2 in the supplementary information).

**Fig. 3:**
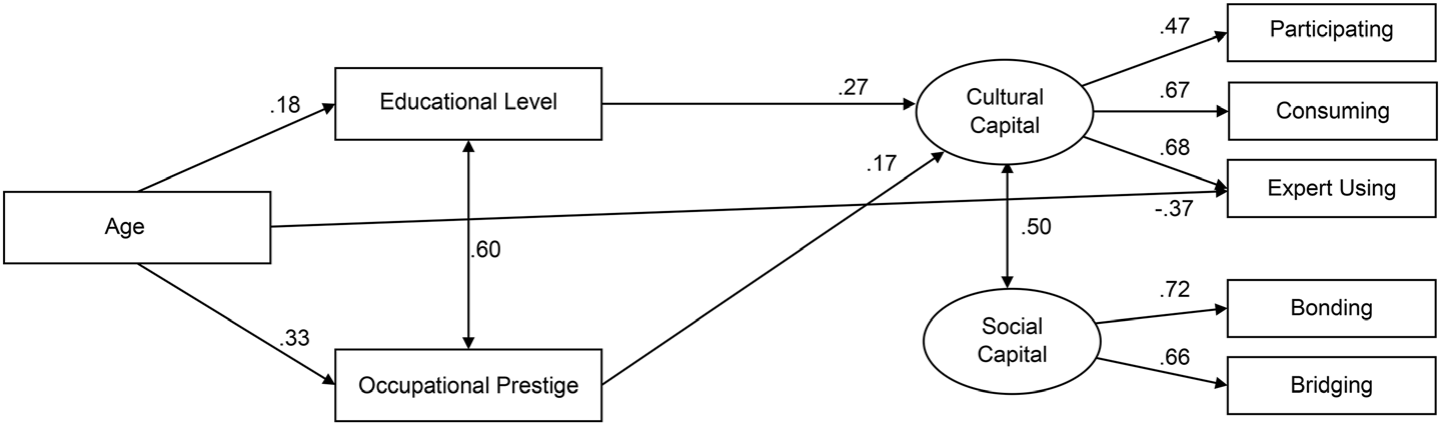
Structural equations model of the relationships between age, educational level, occupational prestige, Cultural Capital, and Social Capital in men (*n* = 389). Values reported are the standardized covariance coefficients (lines with arrows at both ends) and structural coefficients (lines with one arrow) between variables

The resulting model also showed good indices in the men subgroup (*n* = 389): CFI = 0.959; RMSEA = 0.064 [CI = 0.039–0.087]; SRMR = 0.039; *χ*^*2*^_(16)_ = 40.74, *p* < .01, AIC = 10,495 (Fig. 3). Compared to the women’s model, the resulting model did not present the effect of age on Cultural Capital and that of educational level on Social Capital; age had an indirect positive effect only on Cultural Capital through educational level (*β* indirect = 0.05; *p* = .012). Moreover, COVID-19 spread did not show any relationship with the other variables of the model. The explained variance of Cultural Capital was 16%.

Differently from women, for men, the alternative model with a second-order latent variable for Cultural Capital and Social Capital showed a worse fit index (CFI = 0.893; RMSEA = 0.105 [CI = 0.083–0.128]; SRMR = 0.077; *χ*^*2*^_(15)_ = 79.38, *p* < .001; AIC 10,535) as the other alternative models (see Table S3 in the supplementary information).

### Cluster Analysis: Identification of the Optimal Solution of Subgroups Based on their Cultural Capital and Social Capital Level during the Lockdown

Cluster analyses were performed on men, women, and all participants, and similar results were obtained. Therefore, we only presented the results obtained for all participants. Cultural Capital and Social Capital measures were *z*-transformed within all participants to share the same metric. The correlation between Cultural Capital and Social Capital was then performed to exclude multicollinearity that would have impacted the cluster analysis (Hair et al., 2010). We obtained a medium-low correlation coefficient (Pearson’s *r* = .287).

**Fig. 4:**
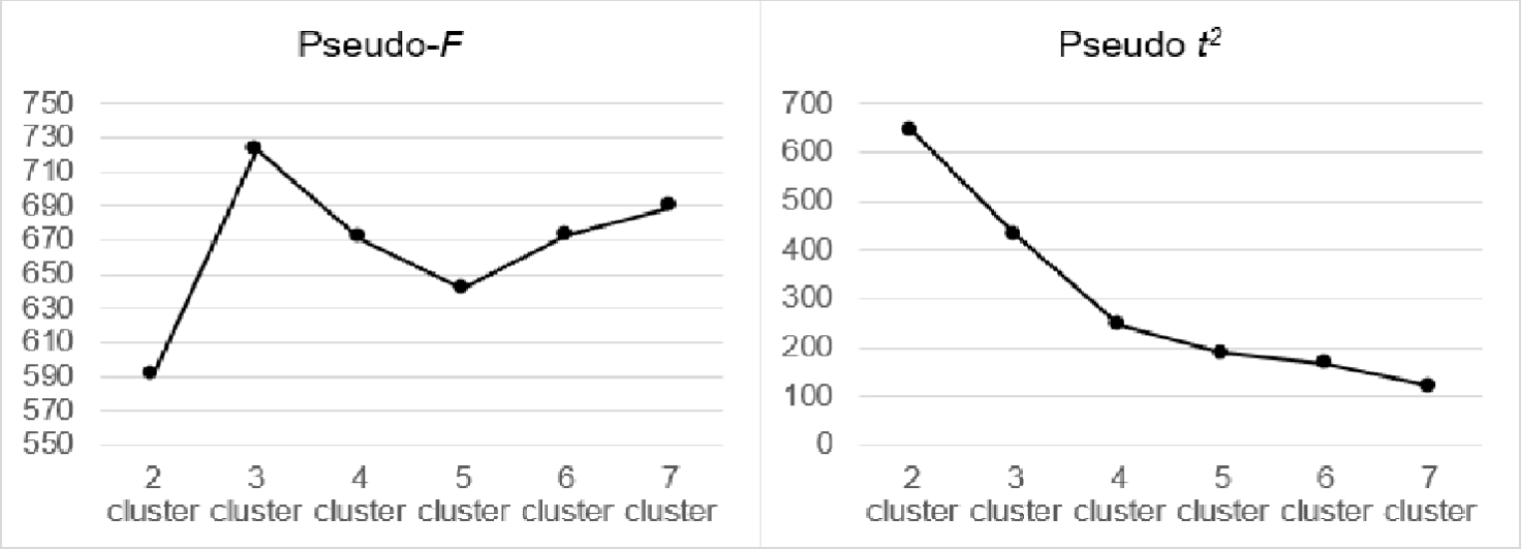
Identification of the subgroups based on their Cultural Capital and Social Capital level during the lockdown: Statistics for the identification of the optimal number of clusters

**Fig. 5:**
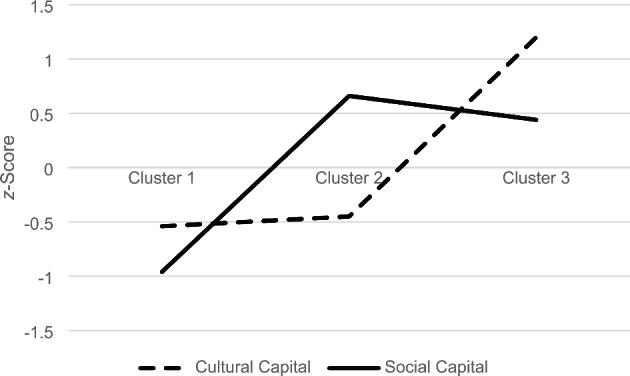
Means of the z-score on the measurement of Cultural Capital and Social Capital for the participants of Clusters 1 (Resourceless), 2 (Mixed), and 3 (Resourceful)

The Ward procedure strongly suggested a three-cluster solution. As shown in Fig. 4, the three-cluster solution had the higher value of pseudo-*F* and pseudo *t*^*2*^ presented a distinct jump for the two-cluster solution, which therefore suggested the three-cluster solution. The complete linkage method and analysis performed on the subgroups confirmed the stability of the Ward’s three-cluster solution. K-means iterative partitioning determined the final cluster memberships. Each cluster exceeded 10% of the participants. Figure 5 shows the profiles of the three clusters. Cluster 1 (*n* = 416), called Resourceless, had a low level of Cultural Capital and Social Capital. Cluster 2 (*n* = 392), called Mixed, had a low level of Cultural Capital and a high level of Social Capital. Cluster 3 (*n* = 317), called Resourceful, presented a high level of both the Capitals.

### Comparisons of the Clusters of Participants

#### Validation of the Three-Cluster Solution

The three clusters differed in terms of educational level (*F*_(2,1122)_ = 24.731, *p* < .001, partial *ƞ*^*2*^ = 0.04) and occupational prestige (*F*_(2,1122)_ = 19.230, *p* < .001, partial *ƞ*^*2*^ = 0.03). Compared to Cluster 3, both Clusters 1 and 2 had a lower educational level (Cluster 1: mean difference = 1.82, Cohen’s *d* = 0.49; Cluster 2: mean difference = 1.47, Cohen’s *d* = 0.41) and occupational prestige (Cluster 1: mean difference = 6.30, Cohen’s *d* = 0.46; Cluster 2: mean difference = 4.10, Cohen’s *d* = 0.30). Additionally, gender differences were found across the three clusters (χ^2^_(2)_ = 7.08, *p* = .029), with more men in Cluster 1 and more women in Cluster 2.

#### Investigation of Differences in the Changes in Cultural Capital and Social Capital During the Lockdown

Statistically significant differences were observed between clusters in Cultural Capital change (*F*_(2, 1122)_ = 60.91, *p* < .001, partial *ƞ*^*2*^ = 0.10) and Social Capital change (*F*_(2, 1122)_ = 39.02, *p* < .001, partial *ƞ*^*2*^ = 0.07). Post-hoc analysis indicated that Cluster 1 had a lower level of Cultural Capital change than Cluster 3 (mean difference = − 0.037, Cohen’s *d* = 0.75), and a lower level of Social Capital change than Cluster 2 (mean difference = − 0.139, Cohen’s *d* = 0.57) and Cluster 3 (mean difference = − 0.131, Cohen’s *d* = 0.35). Cluster 2 had a lower level of Cultural Capital change than Cluster 3 (mean difference = − 0.207, Cohen’s *d* = 0.63). In contrast, the Chi-squared statistic revealed no significant differences between the clusters on the variables concerning the lockdown: COVID-19 spread in the domicile, usual domicile or not, living situation, and number of adults.

## Discussion

The COVID-19 lockdown broke daily routines and, therefore, the way to spend free time, engage in cultural activities, and interact with others. Previous studies have shown that Social Capital was related to the COVID-19 spread (e.g., Alfano 2022) and that Cultural Capital and Social Capital were affected by the COVID-19 pandemic (e.g., Elgar et al., 2020; Modernel & Cornejo, 2022). The present study aimed to investigate (1) how the relationship between Cultural Capital and Social Capital changed in a pandemic context and how the COVID spread was associated with this relationship and (2) whether changes in Cultural Capital and Social Capital during the lockdown were related to the levels of and the relationship between them.

Regarding the first aim, through the structural equation models, we studied the relationships between the participants’ Cultural Capital and Social Capital dimensions, educational level and occupational prestige as indicators of socioeconomic status, age, and the COVID-19 spread in the domicile during the first lockdown in Italy. A relationship with COVID-19 spread was only detected for women: the spread of the pandemic was positively affected by occupational prestige and had a positive relationship with Social Capital. In areas with a greater spread of the virus, women had more prestigious occupations. This probably occurred because the virus was more widespread in Northern Italy (Riccardo et al., 2020), where women have higher occupational prestige than in other areas (Istituto Nazionale di Statistica, 2020b). Similarly, engagement of the women with friends, work/study colleagues, and people in the neighborhood (bonding Social Capital), and with community service or religious groups/associations (bridging Social Capital) may have made the women reluctant to disengage from their human connections, especially if this engagement was already strong before the pandemic (Ding et al., 2020). However, the level of spread of the virus may have pushed women to have more in-person contact with close persons or to be involved in community groups. The lockdown period has been very challenging, and women, which are always more involved in caring and nurturing provision than men (Arber & Ginn, 1995; Christov-Moore et al., 2014; Ferns et al., 2021), have probably been mainly engaged in assistance activities and groups.

This result is at variance with studies that have found that women more than men enact preventive behaviors (Barrios et al., 2021), which in turn resulted associated with a lower prevalence of COVID-19 (Howard et al., 2021; Qian & Jiang, 2022). Yet, the enactment of preventive behaviors is an expression of civil Social Capital (Borgonovi et al., 2021), whereas our study investigated personal Social Capital. Future studies should investigate both forms of Social Capital simultaneously.

Compared to the structural equation model that was recently observed in a pre-pandemic period (Menardo, Viola et al., 2022), in the present study, a U-shaped relationship between age and Cultural and Social Capitals fitted the data better than a linear relationship. We found that middle-aged men have lower Cultural Capital and higher Social Capital than younger and older men, and an inverse pattern was found in women. In middle age, when it is most common to have school-aged children, men prefer to dedicate their free time to enhance their social resources, while women invest more in online cultural activities related to people’s care and assistance (Rania et al., 2022). This result is in line with previous studies suggesting that Social Capital does not follow a linear trend during aging. For example, friendship networks expand during adolescence and young adulthood until they reach stability and shrink during later adulthood (Lambert et al., 2006; Wrzus et al., 2013).

Moreover, in the present study, we found positive instead of negative relationships between Cultural Capital and Social Capital, and a positive effect of educational level on Social Capital only in women. These differences can be attributed mainly to different contexts and participants. Menardo et al.’s investigation was conducted in a university city, with most participants having high levels of education and occupational prestige. In contrast, the present study involved participants from all Italian regions with heterogeneous levels of education and occupational prestige. Interestingly, for women, the alternative structural equation models with a second-order latent variable for Cultural Capital and Social Capital showed similar fit indices of the chosen model without the second-order variable. This result confirmed that these two constructs are highly correlated to form a second-order variable, even during the pandemic lockdown.

This study also aimed to investigate whether changes in Cultural Capital and Social Capital during the first lockdown in Italy were related to the levels of and the relationship between them. To this end, three clusters of participants were identified based on their level of Cultural Capital and Social Capital during the lockdown: (1) low level of Cultural Capital and Social Capital (Resourceless); (2) low level of Cultural Capital and high level of Social Capital (Mixed); and (3) high levels of both the Capitals (Resourceful). These clusters were validated in agreement with the relationships of Cultural Capital and Social Capital with educational level, occupational prestige, and gender (e.g., Menardo, Viola et al., 2022; Nakai, 2011; Van Groenou & Van Tilburg, 2003). Comparisons between the three clusters showed that participants in Cluster 1 faced a worsening of both Cultural Capital and Social Capital; those in Cluster 2 had a deterioration of Cultural Capital and an improvement of Social Capital; and those in Cluster 3 experienced an improvement in both the Capitals.

Previous studies suggested that Social Capital remained stable during the COVID-19 pandemic (Burrmann et al., 2022; Luo et al., 2022). Even if face-to-face social contacts were challenged by social distancing and travel restrictions, social media played a crucial role in allowing people to maintain social relationships (Samutachak et al., 2023). In contrast, other studies showed different trajectories of change in Social Capital (Gallè et al., 2022; Luo et al., 2022).

Our results suggest that the previous level of Social Capital might account for these differences: the lockdown could have contributed to fostering the gap, either exaggerating pre-existing levels of the Capitals or increasing high levels and decreasing low levels. In the present study, people with high levels of Capitals seemed to have used the COVID-19 restrictions to cultivate their cultural interests and be more involved with their network. In contrast, individuals with low levels of Capitals paid the highest price for the social isolation. The lockdown lowered their already low opportunities to use cultural products, attend cultural events, participate in cultural or community service groups, and connect with friends and colleagues. This result agrees with studies that showed an enhancement in cultural consumption during the pandemic only for individuals who already consumed it (Artuso & Palladino, 2021; Feder et al., 2021).

The Cultural Capital and Social Capital are fundamental to healthy behaviors, well-being, and mental health (Giordano & Lindstrom, 2011). Therefore, people with lower levels of cultural and social consumption may have received less information about COVID-19 protection measures and social support from others, with a consequent increase in distress and suffering. This implication is even more relevant considering that no differences between the three clusters have been found regarding the different geographic areas or the characteristics of the lockdown period (i.e., domicile, living situation, and number of adults), which indicates that the present results are generalizable to every type of COVID-19 quarantine experience. It follows that institutions should develop or improve their policies and practices to foster individual resources, and make fairer opportunities available during the pandemic.

This study has inevitable limitations. First, Cultural Capital, Social Capital, educational level and professional prestige are constructs closely linked to the context, i.e., they depend on the characteristics of the community to which one belongs (Coscarelli et al., 2007). Moreover, the first lockdown implemented in Italy was different from strategies to cope with the COVID-19 spread implemented in other countries or later during the pandemic. Consequently, generalization of the results of the present study to those that can be obtained in different countries or during other periods of the COVID-19 pandemic should be done with caution. Moreover, the data collection was based on convenience sampling and, having been carried out during the lockdown, was based only on online strategies. In this way, people without online activities were not involved.

Despite these limitations, the present study is relevant because it demonstrated that the relationships between Cultural Capital, Social Capital, educational attainment, occupational prestige, and age are similar to those identified in a pre-pandemic period. The main results are that only for women the spread of the pandemic has had a positive relationship with their Social Capital and that the lockdown has contributed to increasing the gap between individuals, increasing the high levels, and decreasing the low levels, of both Cultural Capital and Social Capital.

Cultural and Social Capitals encourage healthy behaviors, influence well-being and mental health, help increase community resilience (Paarlberg et al., 2020), and provide critical resources that support people during a crisis (e.g., Barrios et al., 2021; Chen & Meng, 2015). However, studies revealed that during the pandemic, access to cultural offerings and opportunities to interact with others declined for the most vulnerable population (e.g., Modernel & Cornejo 2022). Therefore, in non-pandemic periods institutions should properly consider Cultural and Social Capitals as indicators of the available resources and use social media opportunities (Yum, 2022) to reach the most vulnerable population with awareness campaigns concerning preserving and increasing cultural interests and trustworthy social networks. Furthermore, they could encourage people’s participation by involving community service and local groups/associations to promote the dissemination of knowledge and the adoption of informed behaviors.

## Electronic supplementary material

Supplementary Material
