# Supplementary material for: The Effects of the COVID-19-induced Lockdown on the Social Capital and Cultural Capital in Italy
Source: Soc Indic Res. 2023 Jun 1:1–22. Online ahead of print. doi: 10.1007/s11205-023-03140-7 (PMC10234587; doi:10.1007/s11205-023-03140-7)
Supplement: Supplementary file 1 — Supplementary Material [file 11205_2023_3140_MOESM1_ESM.docx]

**Supplementary Information**

**Table S1**

*Linear and Cubic Function Estimated between Age and Cultural Capital, Social Capital, Educational Level, and Occupational Prestige*

|  | Linear Function | |  | Cubic function | | | |
| --- | --- | --- | --- | --- | --- | --- | --- |
|  | adj *R^2^* | standardize *β* (age) |  | adj *R^2^* | standardize *β* (age) | standardize *β* (age^2^) | standardize *β* (age^3^) |
| Men  *(n = 389)* |  |  |  |  |  |  |  |
| Cultural Capital | -.002 | -.021 |  | .009 | 3.081 | -5.652 | 2.588 |
| Social Capital | -.003 | .006 |  | .010 | -0.031 | 1.075 | -1.065 |
| Educational Level | .035 | .194 |  | .078 | 6.495 | -11.996 | 5.783 |
| Occupational Prestige | .127 | .359 |  | .183 | 6.358 | -10.907 | 4.978 |
| Women  *(n = 736)* |  |  |  |  |  |  |  |
| Cultural Capital | -.001 | -.18 |  | -.002 | -0.784 | 1.325 | -0.568 |
| Social Capital | .001 | .048 |  | .003 | 1.599 | -2.716 | 1.184 |
| Educational Level | .011 | .111 |  | .130 | 8.743 | -15.601 | 7.088 |
| Occupational Prestige | .120 | .348 |  | .196 | 7.009 | -11.922 | 5.350 |

**Table S2**

*Structural Equation Model of the Relationships between Age, Educational Level, Occupational Prestige, Cultural Capital, Social Capital, and Covid Spread Where the Participant Stayed in Women (n = 736): Goodness of Fit Indices of the Chosen and Alternative Models Investigated*

|  | CFI | RMSEA | SRMR | AIC | *χ2*(*df*) |
| --- | --- | --- | --- | --- | --- |
| Chosen model | .949 | .059 (.045-.075) | .037 | 21,452 | 68.41_(19)_*** |
| Alternative models |  |  |  |  |  |
| Model A1 | .911 | .076 (.063-.091) | .047 | 21,488 | 106.13_(20)_******* |
| Model A2 | .952 | .056 (.041-.071) | .036 | 21,448 | 66.17_(20)_******* |
| Model A3 | .910 | .075 (.061-.089) | .047 | 21,488 | 108.05_(21)_******* |

*Note.* These models were obtained by introducing one latent variable in the chosen model, which was measured via educational level and occupational prestige (Model A1), a second-order latent variable for Cultural Capital and Social Capital (Model A2), and both couples of variables (Model A3).

* *p* < .05; ** *p* < .01; *** *p* < .001.

**Table S3**

*Structural Equation Model of the Relationships between Age, Educational Level, Occupational Prestige, Cultural Capital, Social Capital, and Covid Spread Where the Participant Stayed in Men (n = 389): Goodness of Fit Indices of the Chosen and Alternative Models Investigated*

|  | CFI | RMSEA | SRMR | AIC | *χ2*(*df*) |
| --- | --- | --- | --- | --- | --- |
| Chosen model | .959 | .064 (.039-.087) | .039 | 10,495 | 40.74_(16)_** |
| Alternative models |  |  |  |  |  |
| Model A1 | .944 | .072 (.050-.095) | .045 | 10,503 | 51.01_(17)_******* |
| Model A2 | .893 | .105 (.083-.128) | .077 | 10,535 | 79.38_(15)_******* |
| Model A3 | .932 | .081 (.059-.104) | .050 | 10,511 | 56.69_(16)_******* |

*Note.* These models were obtained by introducing one latent variable in the chosen model, which was measured via educational level and occupational prestige (Model A1), a second-order latent variable for Cultural Capital and Social Capital (Model A2), and both couples of variables (Model A3).

* *p* < .05; ** *p* < .01; *** *p* < .001.
